# Conservation and Losses of Non-Coding RNAs in Avian Genomes

**DOI:** 10.1371/journal.pone.0121797

**Published:** 2015-03-30

**Authors:** Paul P. Gardner, Mario Fasold, Sarah W. Burge, Maria Ninova, Jana Hertel, Stephanie Kehr, Tammy E. Steeves, Sam Griffiths-Jones, Peter F. Stadler

**Affiliations:** 1 School of Biological Sciences, University of Canterbury, Christchurch, New Zealand; 2 Biomolecular Interaction Centre, University of Canterbury, Christchurch, New Zealand; 3 Bioinformatics Group, Department of Computer Science; and Interdisciplinary Center for Bioinformatics, University of Leipzig, Härtelstrasse 16-18, D-04107 Leipzig, Germany; 4 ecSeq Bioinformatics, Brandvorwerkstr.43, D-04275 Leipzig, Germany; 5 European Molecular Biology Laboratory, European Bioinformatics Institute, Hinxton, Cambridge, CB10 1SD, UK; 6 Faculty of Life Sciences, University of Manchester, Manchester, United Kingdom; 7 Max Planck Institute for Mathematics in the Sciences, Inselstraße 22, D-04103 Leipzig, Germany; 8 Fraunhofer Institute for Cell Therapy and Immunology, Perlickstrasse 1, D-04103 Leipzig, Germany; 9 Department of Theoretical Chemistry of the University of Vienna, Währingerstrasse 17, A-1090 Vienna, Austria; 10 Center for RNA in Technology and Health, Univ. Copenhagen, Grønnegårdsvej 3, Frederiksberg C, Denmark; 11 Santa Fe Institute, 1399 Hyde Park Road, Santa Fe NM 87501, USA; 12 German Centre for Integrative Biodiversity Research (iDiv) Halle-Jena-Leipzig, Germany; National Institutes of Health, UNITED STATES

## Abstract

Here we present the results of a large-scale bioinformatics annotation of non-coding RNA loci in 48 avian genomes. Our approach uses probabilistic models of hand-curated families from the Rfam database to infer conserved RNA families within each avian genome. We supplement these annotations with predictions from the tRNA annotation tool, tRNAscan-SE and microRNAs from miRBase. We identify 34 lncRNA-associated loci that are conserved between birds and mammals and validate 12 of these in chicken. We report several intriguing cases where a reported mammalian lncRNA, but not its function, is conserved. We also demonstrate extensive conservation of classical ncRNAs (e.g., tRNAs) and more recently discovered ncRNAs (e.g., snoRNAs and miRNAs) in birds. Furthermore, we describe numerous “losses” of several RNA families, and attribute these to either genuine loss, divergence or missing data. In particular, we show that many of these losses are due to the challenges associated with assembling avian microchromosomes. These combined results illustrate the utility of applying homology-based methods for annotating novel vertebrate genomes.

## Introduction

Non-coding RNAs (ncRNAs) are an important class of genes, responsible for the regulation of many key cellular functions. The major RNA families include the classical, highly conserved RNAs, sometimes called “molecular fossils”, such as the transfer RNAs, ribosomal RNAs, RNA components of RNase P and the signal recognition particle [1]. Other classes appear to have have evolved more recently, e.g. the small nucleolar RNAs (snoRNAs), microRNAs (miRNAs) and the long non-coding RNAs (lncRNAs) [2].

The ncRNAs pose serious research challenges, particularly for the field of genomics. For example, they lack the strong statistical signals associated with protein coding genes, e.g. open reading frames, G+C content and codon-usage biases [3].

New sequencing technologies have dramatically expanded the rate at which ncRNAs are discovered and their functions are determined [4]. However, in order to determine the full range of ncRNAs across multiple species we require multiple RNA fractions (e.g. long and short), in multiple species, in multiple developmental stages and tissues types. The costs of this approach are still prohibitive in terms of researcher-time and finances. Consequently, in this study we concentrate on bioinformatic approaches, primarily we use homology-based methods (i.e. covariance models (CMs)). We validate the majority of these predictions using RNA-seq. The CM-based approach that we favour, remain state of the art for ncRNA bioinformatic analyses, as they capture both sequence as well as secondary structure constraints on RNAs [5–7]. This has been shown to improve both the sensitivity and specificity rates for homology assignment [8]. For example, the CM based approach for annotating ncRNAs in genomes requires reliable alignments and consensus secondary structures of representative sequences of RNA families, many of which can be found at Rfam [9–14]. These are used to train probabilistic models that score the likelihood that a database sequence is generated by the same evolutionary processes as the training sequences based upon both sequence and structural information [5–7]. The tRNAscan-SE software package uses CMs to accurately predict transfer RNAs [15, 16].

Independent benchmarks of bioinformatic annotation tools have shown that the CM approaches out-perform alternative methods [8], although their sensitivity can be limited for rapidly evolving families such as vault RNAs or telomerase RNA [17].

The publication of 48 avian genomes, including the previously published chicken [18], zebra finch [19] and turkey [20] with the recently published 45 avian genomes [21–27], provides an exciting opportunity to explore conservation of genomic loci that have been associated with ncRNAs in unprecedented detail.

In the following we explore the conservation patterns of the major classes of avian ncRNA loci in further detail. Using homology search tools and evolutionary constraints, we have produced a set of genome annotations for 48 predominantly non-model bird species for ncRNAs that are conserved across the avian species. This conservative set of annotations is expected to contain the core avian ncRNA loci. We focus our report on the unusual results within the avian lineages. These are either unexpectedly well-conserved ncRNAs or unexpectedly poorly-conserved ncRNAs. The former are ncRNA loci that were not expected to be conserved between the birds and the other vertebrates, particularly those ncRNAs whose function is not conserved in birds. The latter are apparent losses of ncRNA loci expected to be conserved; Here, we consider three categories of such “loss”: First, genuine gene losses in the avian lineage where ncRNAs well conserved in other vertebrates are completely absent in birds. Second, “divergence” where ncRNAs have undergone such significant sequence and structural alternations that homology search tools can no longer detect a relationship between other vertebrate exemplars and avian varieties.

Third, “missing” ncRNAs that failed to be captured in the available, largely fragmented, avian genomes. The avian karyotype is characterized by a large number of chromosomes (average 2*n* ≈ 80) generally consisting of approximately 5 larger “macrochromosomes” and many smaller “microchromosomes” [28–30]. The presence of microchromosomes presents significant assembly challenges [18, 20, 31]. Indeed, of the 48 published avian genomes, 20 of which are high-coverage (> 50*X*), only two were relatively complete chromosomal assemblies when this study was initiated (chicken, zebra finch; [19, 21]) (Chromosomal assemblies of turkey (NCBI GCF_000146605.1) and flycatcher (NCBI GCA_000247815.2) were recently made available). We therefore expect that many ncRNAs in comparative avian genome studies will be missing from the genome assemblies due to microchromosome assembly difficulties.

## Materials and Methods

The 48 bird genome sequences used for the following analyses are available from the phylogenomics analysis of birds website [32, 33].

Bird genomes were searched using the cmsearch program from INFERNAL 1.1 [34, 35] and the covariance models (CMs) from the Rfam database v11.0 [12, 13]. All matches above the curated GA threshold were included. Subsequently, all hits with an E-value greater than 5*x*10^−4^ were discarded, so only matches which passed the Rfam-curated, model-specific GA threshold, and had an E-value smaller than 5*x*10^−4^ were retained. The Rfam database classifies non-coding RNAs into hierarchical groupings. The basic units are “families” which are groups of homologous, alignable sequences; “clans” which are groups of un-alignable (or functionally distinct), homologous families; and “classes” which are groups of clans and families with related biological functions e.g. spliceosomal RNAs, miRNAs and snoRNAs [12]; these categories have been used to classify our results.

In order to obtain good annotations of tRNA genes we ran the specialist tRNA-scan version 1.3.1 annotation tool. This method also uses covariance models to identify tRNAs. However it also uses some heuristics to increase the search-speed, annotates the Isoacceptor Type of each prediction and uses sequence analysis to infer if predictions are likely to be functional or tRNA-derived pseudogenes [15, 16].

Rfam matches and the tRNA-scan results for families belonging to the same clan were then “competed” so that only the best match was retained for any genomic region [12]. To further increase the specificity of our annotations we filtered out families that were identified in < 10% of the avian genomes that we have analyzed in this work. These filtered families largely corresponded to bacterial contamination or species/clade-specific lncRNAs, miRNAs and snoRNAs that have a high evolutionary turn-over (Fig. O in S1 Results) [2, 36, 37].

999 microRNA sequence families, previously annotated in at least one vertebrate, were retrieved from miRBase (v19) [38]. Individual sequences or multiple sequence alignments were used to build covariance models with INFERNAL (v1.1rc3) [34, 35], and these models were searched against the 48 bird genomes, and the genomes of the American alligator and the green turtle as out-groups. Hits with E-value < 10 realigned with the query sequences and the resultant multiple sequence alignments manually inspected and edited using RALEE [39]. Those sequences that did not match the characteristics of a microRNA (conserved seed sequence and hairpin secondary structure) were removed from further analysis.

An additional snoRNA homology search was performed with snoStrip [40]. As initial queries we used deuterostomian snoRNA families from human [41], platypus [42], and chicken [43].

**Fig 1:**
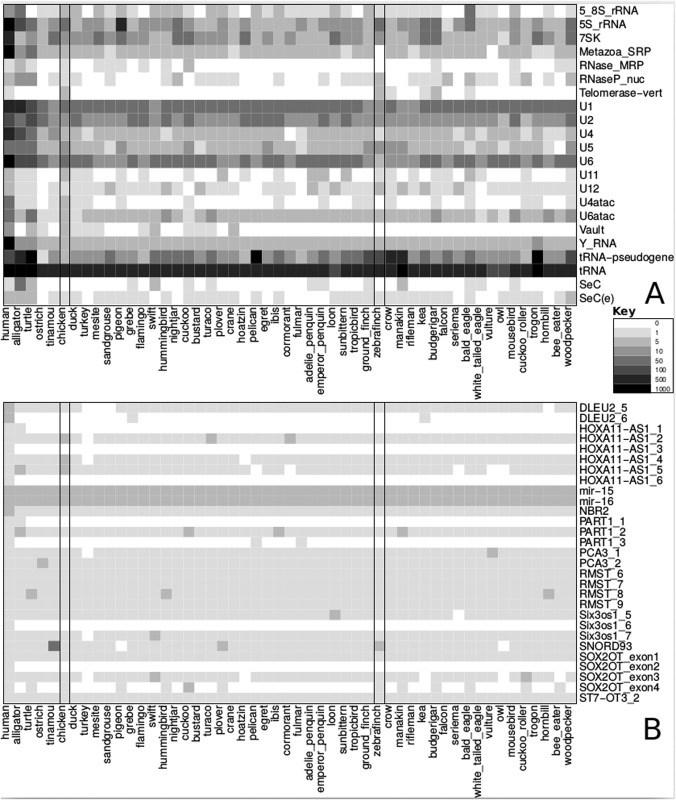
Heatmaps showing the presence/absence and approximate genomic copy-number of “lost, divergent or missing RNAs” and the “unusually, well conserved RNAs”. On the top we show the families that have been identified as surprising RNA losses, divergence or missing data. In several cases functionally related families have also been included, e.g. the RNA components of the major and minor spliceosomes: U1, U2, U4, U5 and U6; and U11, U12, U4atac, U5 and U6atac, respectively. Below we show the unusually, well conserved RNAs, these are predominantly lncRNAs.

The diverse sets of genome annotations were combined and filtered, ensuring conservation in 10% or more of the avian genomes. We collapsed the remaining overlapping annotations into a single annotation. We also generated heatmaps for different groups of ncRNA genes (see Fig. 1 and Figs. A-C in S1 Results). All the scripts and annotations presented here are available from Github [44].

Chicken ncRNA predictions were validated using two separate RNA-seq data sets (IDs are available in Table C in S1 Results). The first data set (Bioproject PRJNA204941) contains 971 million reads and comprises 27 samples from 14 different chicken tissues sequenced on Illumina HiSeq2000 using a small RNA-seq protocol [45]. The second data set (SRA accession SRP041863) contains 1,46 billion Illumina HiSeq reads sequenced from whole chicken embryo RNA from 7 stages using a strand-specific dUTP protocol [45]. The raw reads were checked for quality and adapters clipped if required by the protocol. Preprocessed reads were mapped to the galGal4 reference genome using SEGEMEHL (version 0.1.9) short read aligner [46] and then overlapped with the ncRNA annotations under consideration of strand information.

## Results

There is substantial gain and loss of lncRNAs and other ncRNA associated loci over evolutionary time [2, 36, 37]. It is difficult to assess how many of these “gains” and “losses” are due to limited bioinformatic sequence alignment tools (these generally fail align correctly below 60–50% sequence identity [47]) or due to genuine gains and losses or data missing from the current genome assemblies. Nevertheless, sequence conservation, generally speaking, provides useful evidence for gene and function conservation.

We have identified 66,879 loci in 48 avian genomes that share sequence similarity with previously characterized ncRNAs and are conserved in > 10% of these avian genomes. These loci have been classified into 626 different families, the majority of which correspond to miRNAs and snoRNAs (summarized in Table 1). Out of necessity we have selected a modest number of families for further discussion. These include the lncRNAs that appear to be conserved between Mammals and Aves and the cases of apparent loss of genes that conserved in most other Vertebrates. The supplementary material (S1 Results) contains further discussions of RNA elements.

**Table 1:** A summary of ncRNA genes in human, chicken and all bird genomes. This table contains the total number of annotated ncRNAs from different RNA types in human, the median number for each of the 48 birds and chicken. The number of chicken ncRNA that show evidence for expression is also indicated (the percentage is given in parentheses). The threshold for determining expression was selected based upon a false positive rate of less than 10%.

**ncRNA genes in human, chicken and all bird genomes**				
Number in human	median(48 birds)	Number in chicken	Chicken ncRNAs confirmed with RNA-seq	RNA type
62	25.0	34	12 (35.3%)	Long non-coding RNA
356	499.5	427	280 (65.6%)	microRNA
281	120.0	106	90 (84.9%)	C/D box snoRNA
336	85.5	68	48 (70.6%)	H/ACA box snoRNA
34	13.0	12	12 (100.0%)	Small cajal body RNA
1754	48.5	71	32 (45.1%)	Major spliceosomal RNA
58	3.0	6	3 (50.0%)	Minor spliceosomal RNA
525	82.0	122	88 (72.1%)	Cis-regulatory element
316	6.5	9	3 (33.3%)	7SK RNA
1	0.0	2	0 (0.0%)	Telomerase RNA
9	0.0	2	1 (50.0%)	Vault RNA
892	3.0	3	2 (66.7%)	Y RNA
1084	173.5	300	278 (92.7%)	Transfer RNA
80	9.5	4	2 (50.0%)	Transfer RNA pseudogene
941	3.0	4	2 (50.0%)	SRP RNA
607	7.0	22	10 (45.5%)	Ribosomal RNA
4	1.0	2	2 (100.0%)	RNase P/MRP RNA
7340	1080.0	1194	865 (72.4%)	Total

### Unusually well conserved RNAs

The bulk of the “unusually well conserved RNAs” belong to the long non-coding RNA (lncRNA) group. The lncRNAs are a diverse group of RNAs that have been implicated in a multitude of functional processes [48–51]. These RNAs have largely been characterized in mammalian species, particularly human and mouse and have been shown to be rapidly turned-over by evolutionary processes [37]. Consequently, we generally do not expect these to be conserved outside of Mammals. Notable examples include Xist [52] and H19 [53]. There is emerging evidence for the conservation of “mammalian” lncRNAs in Vertebrates [54, 55]), however, like most lncRNAs, the function of these lncRNAs remains largely unknown. Here, we show the conservation of several lncRNAs that have been well-characterized in humans.

The CM based approach is appropriate for most classes of ncRNA, but the lncRNAs are a particular challenge [50]. CMs cannot model the exon-intron structures of spliced lncRNAs, nor do they deal elegantly with the repeats that many lncRNAs host. Consequently in the latest release of Rfam the lncRNA families that were added were composed of local conserved (and possibly structured elements) within lncRNAs, analogous to the “domains” housed within protein sequences [13]. Whilst some these regions may not reflect functional RNA elements but instead regulatory regions, enhancers or insulators, their syntenic conservation still provides an indication of lncRNA conservation [56].

**Fig 2:**
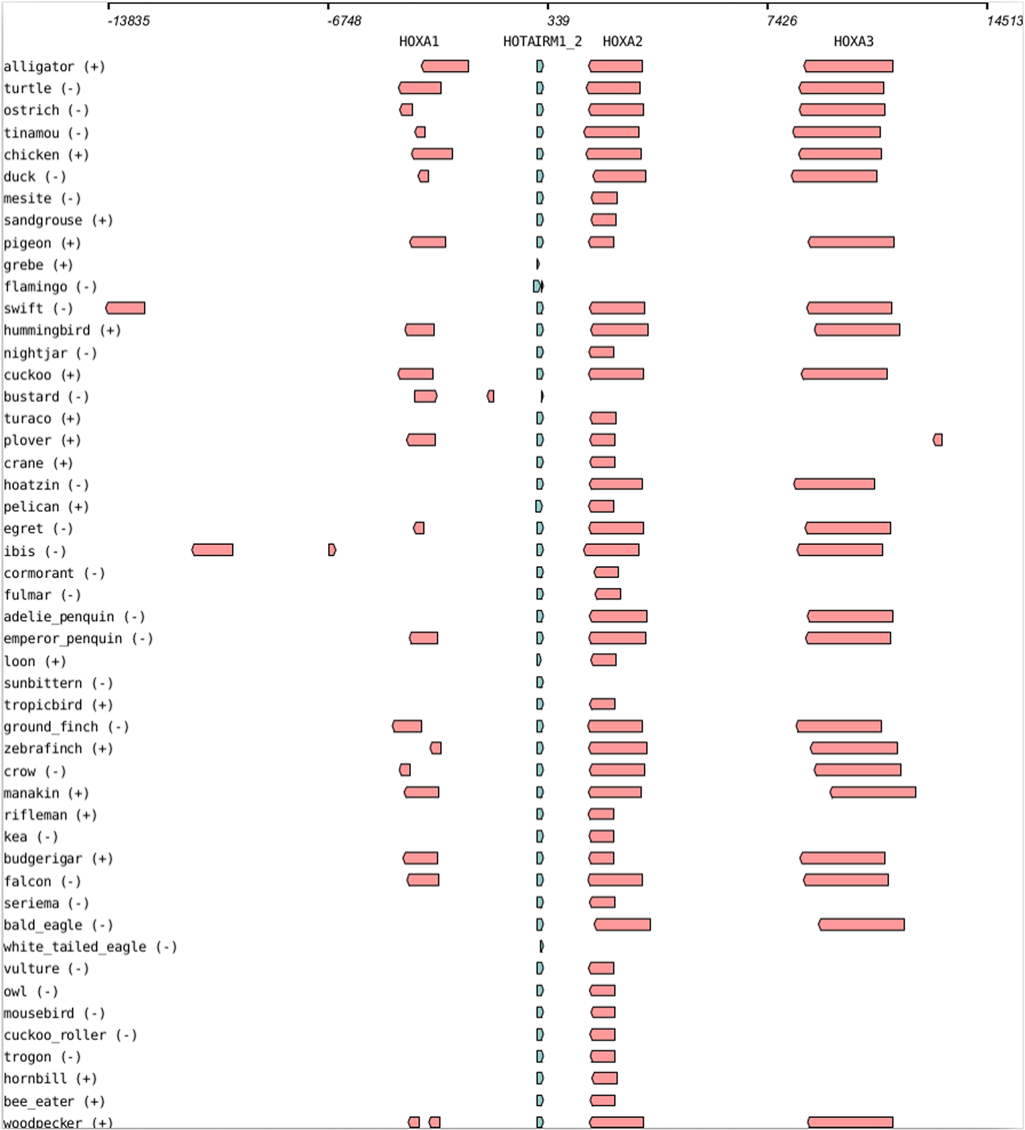
The preservation of gene order (synteny) surrounding the HOTAIRM1 (RF01976) locus across the Avian and other Vertebrate lineages.

When analyzing the RNA-domain annotations it is striking that the order (synteny) of many of the lncRNAs with multiple RNA-domains are consistently preserved in the birds. The annotations of these domains lie in the same genomic region, in the same order as in the mammalian homologs. Thus they support a high degree of evolutionary conservation for the entire lncRNA. In particular the HOXA11-AS1, PART1, PCA3, RMST, Six3os1, SOX2OT and ST7-OT3 lncRNAs have multiple, well conserved RNA-domains (See Fig. 1). The syntenic ordering of these seven lncRNAs and the flanking genes are also preserved between the human and chicken genomes (data not shown). We illustrate this in detail for the HOTAIRM1 lncRNA (see Fig. 2 and Fig. M in S1 Results).

The conservation of these “human” lncRNAs among birds suggests they may also be functional in birds. But what these functions may be is not immediately obvious. For example, PART1 and PCA3 are both described as prostate-specific lncRNAs that play a role in the human androgen-receptor pathway [57–59]. Birds lack a prostate but both males and females express the androgen receptor (AR or NR3C4) in gonadal and non- gonadal tissue [60–63]. Thus, we postulate that PART1 and PCA3 also play a role in the androgen-receptor pathway in birds but whether the expression of these lncRNAs are tissue specific is unknown at present.

The HOX cluster lncRNAs HOTAIRM1 (5 RNA-domains), HOXA11-AS1 (6 RNA-domains), and HOTTIP (4 RNA domains) are conserved across the Mammalian and Avian lineages. In the human genome they are located in the HOXA cluster (hg coordinates chr7:27135743–27245922), one of the most highly conserved regions in vertebrate genomes [64], in antisense orientation between HoxA1 and HoxA2, between HoxA11 and HoxA13, and upstream of HoxA13, respectively. Conservation and expression of HOTAIRM1 and HOXA11-AS1 within the HOXA cluster has been studied in some detail in marsupials [65]. Of the 15 RNA-domains five and six representing all three lncRNAs were recovered in the alligator and turtle genomes. All of them appear in the correct order at the expected, syntenically conserved positions within the HOXA cluster. In the birds, where two or more of the HOX cluster lncRNA RNA-domains were predicted on the same scaffold, this gene order and location within HOX was also preserved.

The RMST (Rhabdomyosarcoma 2 associated transcript) RNA-domains 6, 7, 8, and 9 are conserved across the birds. In each bird the gene order was also consistent with the human ordering. In the alligator and turtle an additional RNA-domain was predicted in each, these were RNA-domains 2 and 4 respectively, again the ordering of the domains was consistent with human. This suggests that the RMST lncRNA is highly conserved. However, little is known about the function of this RNA. It was originally identified in a screen for differentially expressed genes in two Rhabdomyosarcoma tumor types [66].

In addition, the lncRNA DLEU2 is well conserved across the vertebrates, it is a host gene for two miRNA genes, miR-15 and miR-16, both of which are also well conserved across the vertebrates (see Fig. B in S1 Results). DLEU2 is thought to be a tumor-suppressor gene as it is frequently deleted in malignant tumours [67, 68].

The NBR2 lncRNA and BRCA1 gene share a bidirectional promotor [69]. Both are expressed in a broad range of tissues. Extensive research on BRCA1 has shown that it is involved in DNA repair [70]. The function of NBR2 remains unknown, yet its conservation across the vertebrates certainly implies a function (See Fig. 1). We note that the function for this locus may be at the DNA level, however, function at the RNA level cannot be ruled out at this stage.

Of the other classes of RNAs, none showed an unexpected degree of conservation or expansion within the avian lineage. The only exception being the snoRNA, SNORD93. SNORD93 has 92 copies in the tinamou genome, whereas it only has 1–2 copies in all the other vertebrate genomes.

### Unexpectedly poorly conserved ncRNAs: genuine loss, divergence or missing data?

#### Genuine loss

The overall reduction in avian genomic size has been extensively discussed elsewhere [71]. Unsurprisingly, this reduction is reflected in the copy-number of ncRNA genes. Some of the most dramatic examples are the transfer RNAs and pseudogenes which average ∼ 900 and ∼ 580 copies in the human, turtle and alligator genomes, the average copy-numbers of these drop to ∼ 280 and ∼ 100 copies in the avian genomes. In addition to reduction in copy-number, the absence of several, otherwise ubiquitous vertebrate ncRNAs, in the avian lineage are suggestive of genuine gene loss.

Namely, mammalian and amphibian genomes contain three loci of clustered microRNAs from the mir-17 and mir-92 families [72]. One of these clusters (cluster II, with families mir-106b, mir-93 and mir-25) was not found in turtles, crocodiles and birds (see Fig. F in S1 Results). In addition, the microRNA family let-7 is the most diverse microRNA family with 14 paralogs in human. These genes also localize in 7 genomic clusters, together with mir-100 and mir-125 miRNA families (see previous study on the evolution of the let-7 miRNA cluster in [73]). In Sauropsids we observed that cluster A—which is strongly conserved in vertebrates has been completely lost in the avian lineage. Another obvious loss in birds is cluster F, containing two let-7 microRNA paralogs. Cluster H, on the other hand has been retained in all oviparous animals and completely lost later, after the split of Theria (see Fig. G in S1 Results).

#### Divergence

In order to determine to what extent the absence of some ncRNAs from the infernal-based annotation is caused by sequence divergence beyond the thresholds of the Rfam CMs, we complemented our analysis by dedicated searches for a few of these RNA groups. Our ability to find additional homologs for several RNA families that fill gaps in the abundance matrices (Fig. 1) strongly suggests that conspicuous absences, in particular of LUCA and LECA RNAs, are caused by incomplete data in the current assemblies and sequence divergence rather then genuine losses.

Vertebrate Y RNAs typically form a cluster comprising four well-defined paralog groups Y1, Y3, Y4, and Y5. In line with [74] we find that the Y5 paralog family is absent from all bird genomes, while it is still present in both alligator and turtle (see Fig. D in S1 Results). Within the avian lineage, we find a conserved Y4-Y3-Y1 cluster. Apparently, broken-up clusters are in most cases consistent with breaks (e.g. ends of contigs) in the available sequence assemblies. In several genomes we observe one or a few additional Y RNA homologs unlinked to the canonical Y RNA cluster. These sequences can be identified unambiguously as derived members of one of the three ancestral paralog groups, they almost always fit less well to the consensus (as measured by the CM bit score of paralog group specific covariance models) than the paralog linked to cluster, and there is no indication that any of these additional copies is evolutionarily conserved over longer time scales. We therefore suggest that most or all of these interspersed copies are in fact pseudogenes (see below).

#### Missing data

Seven families of “core” ncRNAs were found in some avian genomes but not others (Fig. 1). These families range in conservation level from being ubiquitous to cellular-life (RNase P and tRNA-sec), present in most Bilateria (vault), present in the majority of eukaryotes (RNase MRP, U4atac and U11) and present in all vertebrates (telomerase) [2]. Therefore, the genuine loss or even diversification of these ncRNA families in the avian lineage is unlikely. Rather, this lack of phylogenetic signal, combined with the fragmented nature of the vast majority of these genomes described above (i.e., of the 48 avian genomes, only the chicken and zebra finch were chromosomally assembled [19, 21] when this project was initiated), suggests the most likely explanation is that these ncRNA families are indicative of missing data. Indeed, of the seven missing ncRNA families, six where found in the chicken genome and three were found in the zebra finch genome. Furthermore, only one of these (RNase MRP) is found on a chicken macrochromosome, and all remaining missing ncRNAs are found on chicken microchromosomes (see Table A in S1 Results). A Fisher's exact test showed that there are significantly more missing ncRNAs on microchromosomes than macrochromosomes, *P* < 10^16^ (we use the micro/macro-chromosome assignments from the chicken genome as this is the most complete avian genome). Thus, we suggest that many of these ncRNAs families are missing because: (1) they are predominantly found on microchromosomes [this study] and (2) the vast majority of avian microchromosomes remain unsequenced [21, 31]. Furthermore, there has been minimal chromosomal rearrangement across the avian genome [21]. Therefore, it is likely that the chicken microchromosomal genes are also on microchromosomes in the other avians.

To wit, we performed dedicated searches for a selection of these missing ncRNA families. Here, tRNAscan is tuned for specificity and thus misses several occurrences of tRNA-sec that are easily found in the majority of genomes by blastn with *E* ≤ 10^−30^. In some cases the sequences appear degraded at the ends, which is likely due to low sequence quality at the very ends of contigs or scaffolds. A blastn search also readily retrieves additional RNase P and RNAse MRP RNAs in the majority of genomes, albeit only the best conserved regions are captured. In many cases these additional candidates are incomplete or contain undetermined sequence, which explains why they are missed by the CMs [75, 76].

### Classic RNAs: LUCA and LECA

Many RNA families constitute the most evolutionarily conserved genes across all life on this planet [1]. Examples of RNAs derived from the Last Universal Common Ancestor (LUCA) include the transfer RNAs (tRNA), ribosomal RNAs (rRNA), RNA components of RNase P (RNase P RNA), RNase MRP (RNase MRP RNA) and the signal recognition particle (SRP RNA). Other classes of RNA are likely to have been components of the Last Eukaryotic Common Ancestor (LECA). These include the telomerase RNA, major spliceosomal RNAs (U1, U2, U4, U5, and U6) and the minor spliceosomal RNAs (U11, U12, U4atac, and U6atac) [2].

Unsurprisingly, the bulk of these classes of RNAs are well represented across the bird genomes (See Fig. 1). However, there appear to have been “losses” of a few of these RNAs in certain bird species. Some of these may be due to sequence divergence, of which there are several notable examples e.g. [77–81]. Other apparent loss may be explained by incomplete genome coverage.

A number of the classic RNAs are incorporated into RNA-protein complexes (RNPs) involved in core cellular processes. An example of this are the spliceosomal RNAs. Based upon the presence/absence patterns of the major spliceosomal RNAs they are all well represented in these genome sequences. The exceptions to this observation are the U4 RNA in cormorant and the U5 RNA in the bee eater which are both missing. These two genomes are low coverage, suggesting these genes weren’t captured in the current assembly. The minor spliceosomal RNAs are more interesting, the U4atac and U11 snRNAs show widespread patterns of loss, even in some of the high coverage genomes. These RNAs are frequently missed in bioinformatic screens. Indicating either frequent loss [82] or sequences that have diverged beyond the ability of detection by covariance models [83].

The telomerase RNA is also largely missing from the avian annotations. This RNA acts as a template for the telomerase enzyme that extends the telomeres found on chromosome ends. It is only found in the chicken, bald eagle, kea, budgerigar, crow and zebrafinch. Homology searches searches with the telomerase reverse transcriptase (TERT) protein show that the protein component of the telomerase RNP is conserved across all the bird genomes (data not shown). This pattern of presumably divergent telomerase RNA and conserved telomerase protein has been noted previously, most notably in the fungi [77, 78].

The RNA components of RNase P and RNase MRP also appear to have undergone dramatic losses within the bird lineage. RNase P is required for the maturation of tRNA, the paralogous enzyme, RNase MRP is required for the maturation of rRNA. Each RNP cleaves smaller RNAs from larger transcripts [84]. It is unlikely that the these genes have been lost in any of the birds. Homology searches with the RNase associated protein coding genes (POP1, POP4, POP5, POP7, RPP1, RPP14, RPP25, RPP38, RPP40 and RPR2), identified viable homologs of each in all of the bird genomes [85] (data not shown). This suggests that the bird RNase P and MRP RNAs may have diverged slightly from the canonical models.

The 5.8S component of the ribosome in the turtle, turkey bustard, hoatzin, flamingo, tropicbird, seriema, owl, cuckoo roller, trogon, bee eater and falcon appears to have been lost (See Fig. 1). The rRNA repeats are frequently not assembled, consequently it is not surprising to see “losses” in these [86]. Furthermore, the genomes for these species are also low-coverage.

### Small nucleolar RNAs

Small nucleolar RNAs (snoRNAs) are important ncRNAs that participate in the maturation of other functional RNAs [87]. The bulk of the characterised snoRNAs guide either methylation or pseudouridylation modifications, primarily of rRNAs but also spliceosomal RNAs. The two types of modifications are guided by two different types of RNA, the box C/D and the H/ACA snoRNAs respectively, each with a characteristic cohort of motifs and secondary structures [88].

There are 66 ribosomal modification sites, guided by 59 snoRNA families, that are preserved between *H. sapiens* and *S. cerevisiae* [41]. Of these, 45 snoRNA families are conserved in the bird data set. Over a third of the apparent losses of the yeast-human conserved snoRNA families appear to cluster on 2 loci of the ancestral vertebrate genome. We investigated these losses further.

The first cluster is found at chr11:62620797–62622484 on the human genome (hg19) and contains SNORD27, SNORD29 and SNORD31 of the human-yeast conserved snoRNAs. These snoRNAs are located in the inside-out gene SNHG1 which hosts a total of eight C/D box snoRNAs: SNORD25, SNORD26, SNORD27, SNORD28, SNORD29, SNORD22, SNORD30 and SNORD31 [89]. Each of which are also found in the alligator and turtle genomes within a 3–4 KB locus, yet these have largely been lost in the birds. However, five of the eight snoRNAs are located in the tinamou genome. These are located on the same scaffold and are within 2 KB of each other. This implies that SNHG1 is conserved in the tinamou. Loci with four of the eight snoRNAs can be found in zebrafinch, ground-finch, and bald eagle. Still, three of the eight are located in the ostrich, crow, and cuckoo genomes, again within 2 KB of each other on the same scaffolds. This complex pattern of loss could be attributed to many different models, e.g. multiple losses in birds, poor homology modelling or incomplete genome sequences.

The second cluster is located at chr19:49993222–49994231 on the human genome (hg19) and contains two copies of SNORD33 and one SNORD34 all within a 1 KB genomic region. The turtle and alligator genomes retain the two copies of SNORD33 yet don’t have an obvious SNORD34 gene on the same scaffold. Within the bird genomes, the crow and rifleman each retain a single SNORD33 and SNORD34 gene on the same scaffold. While the ground-finch and bald eagle retain a single SNORD33 and the zebrafinch and seriema retain a single SNORD34 (see Fig. C in S1 Results). In human these snoRNAs are intronic to the host gene, ribosomal protein L13a (RPL13A). Based on BLASTP (version 2.2.18) homology searches for the RPL13A gene, the protein is conserved in the human and turtle genomes and in the bald eagle, crow, rifleman and zebrafinch avian genomes (data not shown). Therefore the RPL13A gene and corresponding intronic snoRNAs show the same conservation pattern. This supports a pattern of loss of the RPL13A gene and the intronic snoRNAs that it hosts in the bird genomes.

### MicroRNAs

MicroRNAs are an important class of non-coding RNA. They have been found in the genomes of Chromalveolata [90, 91], Metazoa [92–94], Mycetozoa [95, 96], Viridiplantae [97–100] and Viruses [101–104]. The miRNAs have been shown to regulate the expression of large numbers of messenger RNAs [105]. The mature miRNA product is generally 22 nucleotides long which is usually processed from a larger RNA that is characterised by a stable hairpin-shaped secondary structure.

Chicken and zebrafinch are the only birds with previously annotated microRNAs. We searched for homologs of these and other vertebrate microRNAs in the genomes of the 48 birds, American alligator and green turtle. Overall, we annotate a total of 16617 putative microRNA loci, homologous to 543 known microRNA genes, of which 487 are annotated in chicken and/or zebra finch, while 56 have been so far known only in non-avian vertebrates. The numbers of annotated loci in the individual species are approximately equal—300–400 per species, except for the turkey (Meleagris gallopavo) where we identified 543 sequences homologous to known microRNAs.

In addition, we can confidently identify a further 3 microRNA families that are present in mammals, and turtle and/or crocodile, but not in any avian genome (mir-150, mir-208, mir-590). This suggests that these sequences were lost in the last common ancestor of archosaurs or birds. There are also a number of microRNAs that are predicted to be present in turtles and/or crocodiles, and only a small number of bird genomes. Indeed, there are many missing annotations, species-specific and otherwise, that are not consistent with the consensus phylogeny, and could be due to either incomplete genomes or widespread microRNA loss.

The turkey genome contains a high number (190) of microRNAs so far found only in chicken, which account for the higher number of annotated sequences in this genome compared with other birds. This is consistent with its phylogenetic position as the closest chicken relative among the examined birds. However, 101 chicken microRNAs have no homolog in the turkey or other bird genomes, suggesting that these genes are chicken-specific. This is consistent with previous reports of large number of species specific microRNAs in all animals, and supports the view of fast microRNA turnover during animal evolution [2].

### Cis-regulatory elements

The cis-regulatory RNAs are a group of RNA structures encoded on mRNAs. Generally they are involved in regulating the expression of the mRNA they are encoded within. Others may recode the translated protein product into an alternate sequence.

This group includes the iron response element (IRE) [106] and the histone 3^′^ UTR (histone3) [107]. These are structured motifs bound by regulatory proteins. The selenocysteine insertion sequence (SECIS) is a structured motif that recodes UGA stop codons to selenocysteines [108] and the GABRA3 stem-loop is a structure recognised by the ADAR enzyme family. This enzyme edits adenine nucleotides to inosine, in this case recoding an isoleucine codon to methionine in exon 9 of the GABRA3 gene [109].

These regulatory elements and others, including an internal ribosome entry site (IRES), potassium channel RNA editing signal (K chan RES), Antizyme RNA frameshifting stimulation element (Antizyme FSE), vimentin 3^′^ UTR protein-binding region (Vimentin3) and a connective tissue growth factor (CTGF) 3^′^ UTR element (CAESAR) are conserved across a diverse group of vertebrates, including the bird lineages explored here (See Fig. 1).

### Pseudogenes

Non-coding RNA derived pseudogenes are a major problem for many ncRNA annotation projects. The human genome, for example, contains > 1 million Alu repeats, which are derived from the SRP RNA [110]. The existing Rfam annotation of the human genome, in particular, contains a number of problematic families that appear to have been excessively pseudogenised. The U6 snRNA, SRP RNA and Y RNA families have 1,371, 941 and 892 annotations in the human genome. These are a heterogenous mix of pseudogenised, paralogous, diverged or functional copies of these families. Unfortunately, a generalized model of RNA pseudogenes has not been incorporated into the main covariance model package, Infernal. An approach used by tRNAscan [15], is, in theory, generalizable to other RNA families but this remains a work in progress.

It is possible that the avian annotations also contains excessive pseudogenes. However, it has previously been noted that avian genomes are significantly smaller than other vertebrate species [18]. We have also noted a corresponding reduction in the number of paralogs and presumed ncRNA-derived pseudogenes in the avian genomes (see Fig. L in S1 Results). The problematic human families, U6 snRNA, SRP RNA and Y RNA have, for example, just 26, 4 and 3 annotations respectively in the chicken genome and 13, 3 and 3 annotations respectively, on average, in the 48 avian genomes used here. Therefore, we conclude that the majority of our annotations are in fact functional orthologs.

### Experimentally confirmed ncRNAs

The ncRNAs presented here have been identified using homology models and are evolutionarily conserved in multiple avian species. In order to further validate these predictions we have used strand-specific total RNA-seq and small RNA-seq of multiple chicken tissues. After mapping the RNA-seq data to the chicken genome (see Methods for details), we identified a threshold for calling a gene as expressed by limiting our estimated false-positive rate to approximately 10%. This FDR was estimated using a negative control of randomly selected, un-annotated regions of the genome. Since some regions may be genuinely expressed, the true FDR is potentially lower than 10%. Overall, the number of ncRNAs we have identified in this work that are expressed above background levels is 865 (72.4%) (see Table 1). This shows that 7.0 times more of our ncRNA predictions are expressed than is expected by chance (Fisher’s exact test: *P* < 10^16^). This number is an underestimate of the fraction of our annotations that are genuinely expressed, as only a fraction of the developmental stages and tissues of chicken have been characterized with RNA-seq. Furthermore, some ncRNAs are expressed in highly specific conditions [111, 112].

The classes of RNAs where the majority of our annotations were experimentally confirmed includes microRNAs, snoRNAs, cis-regulatory elements, tRNAs, SRP RNA and RNase P/MRP RNA. The RNA-seq data could not provide evidence for a telomerase RNA transcript, which are only generally only expressed in embryonic, stem or cancerous tissues. Only a small fraction of the 7SK RNA, the minor spliceosomal RNAs and the lncRNAs could be confirmed with the 10% FDR threshold. There are a number of possible explanations for this: the multiple copies of the 7SK RNA may be functionally redundant and can therefore compensate for one another; The minor spliceosome is, as the name suggests, a rarely used alternative spliceosome; and the lncRNAs are generally expressed at low levels under specific conditions [111, 113]. Nevertheless, 12 of the 34 lncRNA-associated Rfam models were found to be expressed, these included HOTAIRM1, HOXA11-AS1, NBR2, SOX2OT and ST7-OT3 (see Fig. M in S1 Results for an illustration of RNA expression at the HOTAIRM1 locus).

## Discussion

In this work we have provided a comprehensive annotation of non-coding RNAs in genome sequences using homology-based methods. The homology-based tools have distinct advantages over experimental-based approaches as not all RNAs are expressed in any particular tissue-type or developmental-stage, in fact some RNAs have extremely specific expression profiles, e.g. the lsy-6 microRNA [112]. We have identified previously unrecognized conservation of ncRNAs in avian genomes and some surprising “losses” of otherwise well conserved ncRNAs. We have shown that most of these losses are due to difficulties assembling avian microchromosomes rather than *bona fide* gene loss. A large fraction of our annotations have been confirmed using RNA-seq data, which also showed a 7-fold enrichment of expression within our annotations relative to unannotated regions.

The collection of ncRNA sequences is generally biased towards model organisms [2, 87]. However, we have shown that using data from well studied lineages such as mammals can also result in quality annotations of sister taxa such as Aves.

In summary, these results indicate we are in the very early phases of determining the functions of many RNA families. This is illustrated by the fact that the reported functions of some ncRNAs are mammal-specific, yet these are also found in bird genomes.

## Supporting Information

S1 Results Supplementary results and discussion. Further results and discussion of poorly conserved ncRNAs, genomic contamination, additional analyses and the sources of avian sequencing data.
(PDF)
