# Supplementary material for: Conservation and Losses of Non-Coding RNAs in Avian Genomes
Source: PLoS One. 2015 Mar 30;10(3):e0121797. doi: 10.1371/journal.pone.0121797 (PMC4378963; doi:10.1371/journal.pone.0121797)
Supplement: S1 Results — Further results and discussion of poorly conserved ncRNAs, genomic contamination, additional analyses and the sources of avian sequencing data. (PDF) [file pone.0121797.s001.pdf]

Supplementary material for: Conservation and losses of non-coding RNAs in avian genomes

Paul P. Gardner*[1,2] , Mario Fasold[3,4] , Sarah W. Burge[5] , Maria Ninova[6] , Jana Hertel[3] , Stephanie Kehr[3] , Tammy E. Steeves[1] , Sam Griffiths-Jones[6] and Peter F. Stadler[3,7–11]

[1] School of Biological Sciences, University of Canterbury, Private Bag 4800, Christchurch, New Zealand. [2] Biomolecular Interaction Centre, University of Canterbury, Private Bag 4800, Christchurch, New Zealand. [3] Bioinformatics Group, Department of Computer Science; and Interdisciplinary Center for Bioinformatics, University of Leipzig, Härtelstrasse 16-18, D-04107 Leipzig, Germany. [4] ecSeq Bioinformatics, Brandvorwerkstr.43, D-04275 Leipzig, Germany. [5] European Molecular Biology Laboratory, European Bioinformatics Institute, Hinxton, Cambridge, CB10 1SD, UK. [6] Faculty of Life Sciences, University of Manchester, Manchester, United Kingdom. [7] Max Planck Institute for Mathematics in the Sciences, Inselstraße 22, D-04103 Leipzig, Germany. [8] Fraunhofer Institute for Cell Therapy and Immunology, Perlickstrasse 1, D-04103 Leipzig, Germany. [9] Department of Theoretical Chemistry of the University of Vienna, Währingerstrasse 17, A-1090 Vienna. [1]0 Center for RNA in Technology and Health, Univ. Copenhagen, Grønnegårdsvej 3, Frederiksberg C, Denmark. [1]1 Santa Fe Institute, 1399 Hyde Park Road, Santa Fe NM 87501, USA. [1]0 German Centre for Integrative Biodiversity Research (iDiv) Halle-Jena-Leipzig.

Email: Paul P. Gardner*- paul.gardner@canterbury.ac.nz; mario@bioinf.uni-leipzig.de; swb@ebi.ac.uk; Maria.Ninova@postgrad.manchester.ac.uk; jana@bioinf.uni-leipzig.de; steffi@bioinf.uni-leipzig.de; tammy.steeves@canterbury.ac.nz; sam.griffiths-jones@manchester.ac.uk; studla@bioinf.uni-leipzig.de;

*To whom correspondence should be addressed

Supplementary results

In the following we explore in further detail the results that were not discussed in the main manuscript.

Unexpectedly poorly conserved ncRNAs: testing divergence

In order to get an idea to what extent the absence of these RNAs from the `infernal-based` annotation is caused by sequence divergence beyond the thresholds of the Rfam CMs and/or missing or incomplete data, we complemented our analysis by dedicated searches for a few of these RNA groups.

The simplest case are the selenocysteine tRNAs. Here, tRNAscan is tuned for specificity and thus misses several occurrences that are easily found by `blastn` with $E \leq 10^{-30}$. In some cases the sequences appear degraded at the ends, which may be explained e.g. by low sequence quality at the very ends of contigs or scaffolds. A `blastn` search also readily retrieves additional RNase P and RNAse MRP RNAs, capturing only the best conserved regions. In many cases these additional candidates are incomplete or contain undetermined sequence, explaining why they are missed by the CMs. Overall, we identify tRNA-Sec in

most and RNAse P and MRP RNAs in the majority of the genomes. An additional candidate could also be retrieved for telomerase RNA. Telomerase is well known to exhibit very poor sequence conservation and rapid variations in size that make it notoriously hard to identify by homology search [1]. The poor return thus does not come as a surprise. Vault RNA homology searches with `blastn` remained unsuccessful, therefore we constructed a sauropsid-specific CM. In addition to the hits identified by the Rfam model we obtained three additional homologs. Vault RNAs, with a size of about 100 nt, exhibit conserved sequence patterns only at their ends, with essentially unconstrained sequence in the central part. Their identification is one of the well-known and difficult problems for homology search [2, 3].

Our ability to find additional homologs for several RNA families that fill gaps in the abundance matrices (Figure A) strongly suggests that conspicuous absences, in particular of LUCA and LECA RNAs, are caused by incomplete data in the current assemblies and sequence divergence rather then true losses.

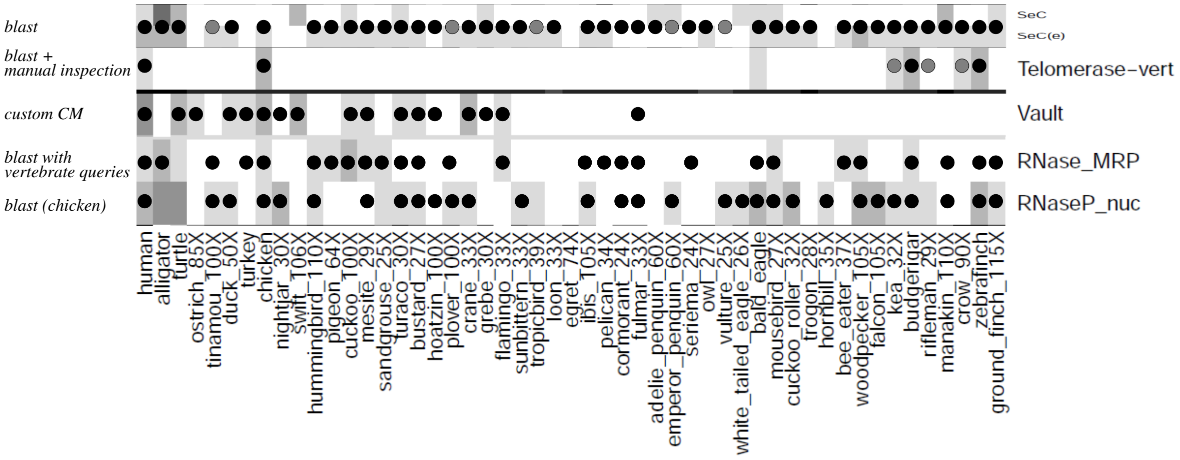

Figure A: Additional homologs of some sparsely represented RNA families were discovered using dedicated search strategies combined with highly sensitive settings, synteny information, lineage-specific CMs and subsequent manual inspection.

Exceptional RNAs

A number of other ncRNAs can also be found in Eukaryotic genomes. These do not fit into the main classes of RNA but still perform vital roles in the function and evolution of Eukaryotes. Their functions are diverse and many have not yet been characterised.

An example of an uncharacterised RNA is the ultraconserved element, uc.338 (also known as TUC338) [4–6]. The uc.338 element is derived from a short interspersed element (SINE) called the lobe-finned fishes SINE (LF-SINE) as it is conserved between the coelacanth and mammals [5]. Analysis of

the expression of uc.338 implies that it plays a role in the progression of hepatocellular carcinoma, possibly by influencing cell growth [6]. This RNA is conserved in the birds and appears to have been duplicated in several lineages.

The Y RNA is an enigmatic ncRNA where we know very little about the function. It was discovered in the 1980s in ribonucleoprotein complexes [7]. The function of the Y RNAs remain unknown, but evidence is emerging that they may be associated with DNA replication [8]. There are 4 functional Y RNAs encoded in the human genome, Y1, Y3, Y4 and Y5. However, there are hundreds of pseudogenised copies of the Y RNA scattered throughout the human genome [9]. In the birds and other lizards, we identify between two and seven Y RNA paralogs (See Figure B).

The Vault RNA forms a major component of the vault ribonucleoprotein complex, this is one of the largest particles found in the vertebrate cell; In fact, it is larger than the ribosome [10]. As yet not much is known about the function of Vault. The Vault RNA has been shown to be broadly conserved in metazoans [11]. However, in the bird lineages it appears to have either been lost or diversified.

Contamination

Bacterial families can be used to identify problematic sequences that are likely to be the result of contamination from non-avian sources. We identified a number of RNA families of bacterial origin in the avian genomes. These have been reported and will be dealt with in later updates to the avian genome sequences. Contamination partially explains the large number of low numbers of some families, high-evolutionary turnover explains most of the remainder (See Supplementary Figure O) [12–14].

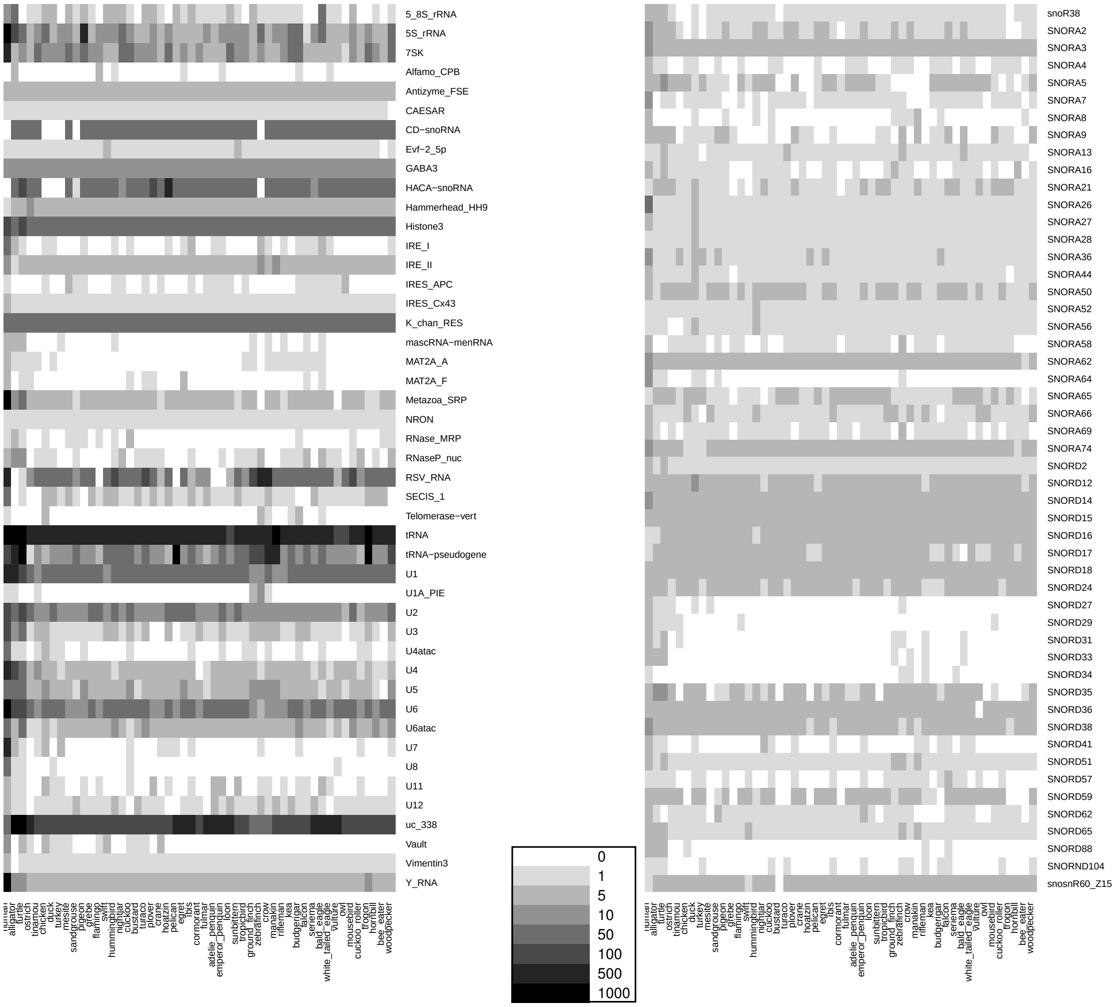

Figure B: This figure illustrates the number of copies of Rfam and tRNAscan annotations for each RNA family in each avian species and outgroups. The families are ordered alphabetically vertically and the species are in phylogenetic order horizontally. Darker shades indicate high copy numbers, lighter shades indicate fewer, white indicates no predictions were made.

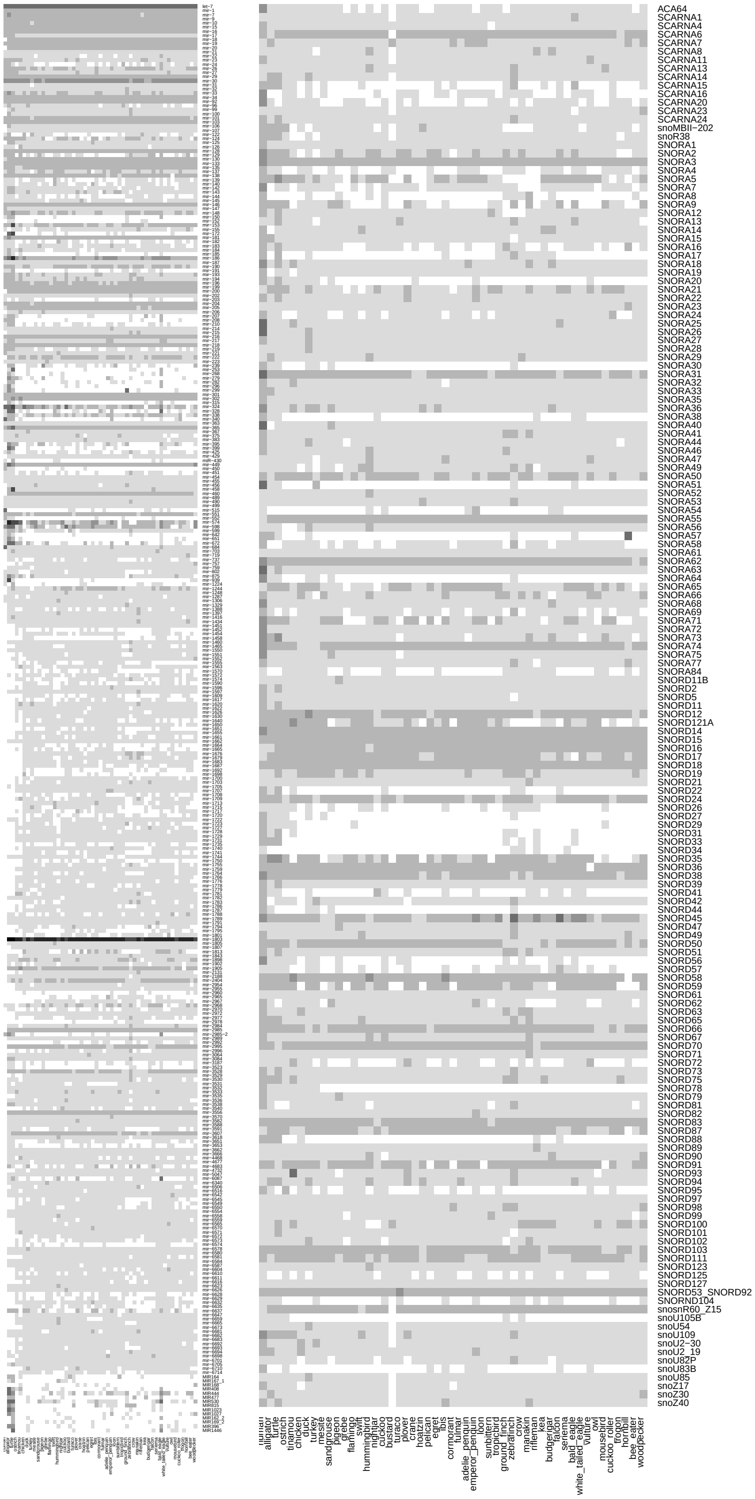

Figure C: Heatmaps showing the presence/absence and approximate copy number of **miRNA** families on the right and **snoRNA** families on the left.

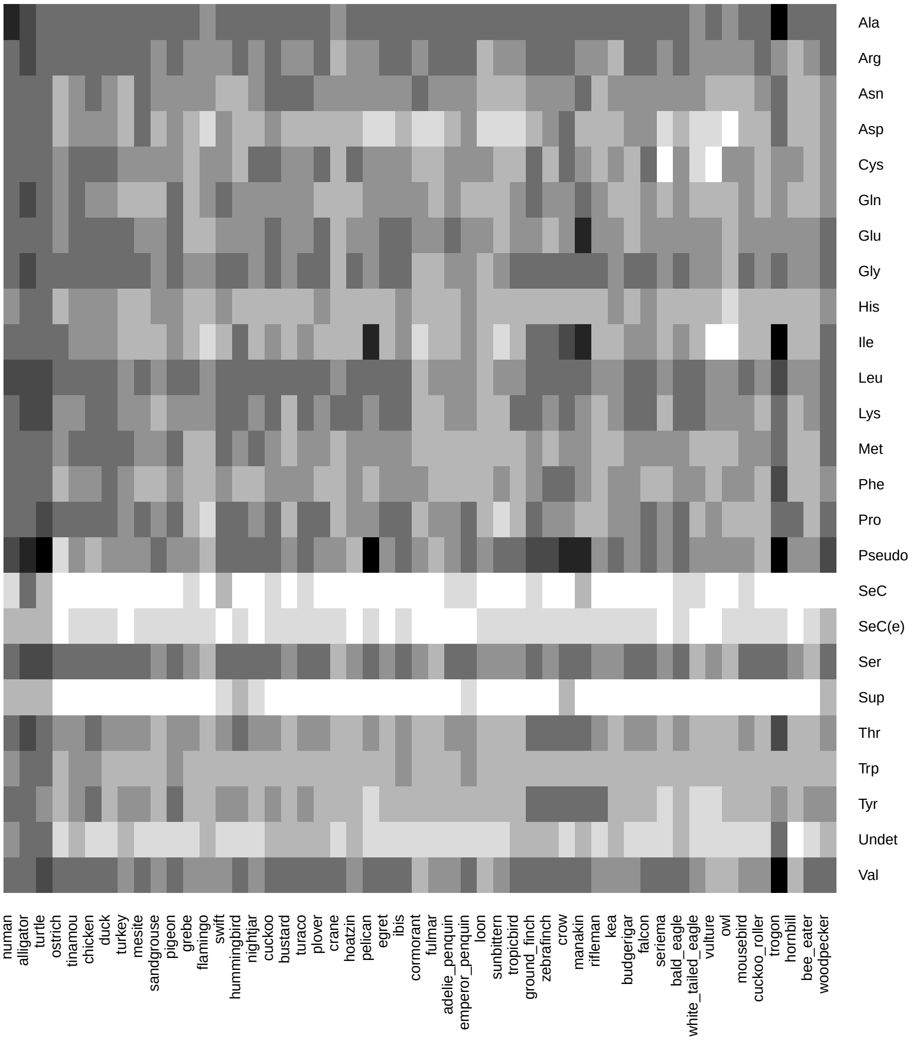

Figure D: Heatmaps showing the presence/absence and approximate copy number of **tRNA** families.

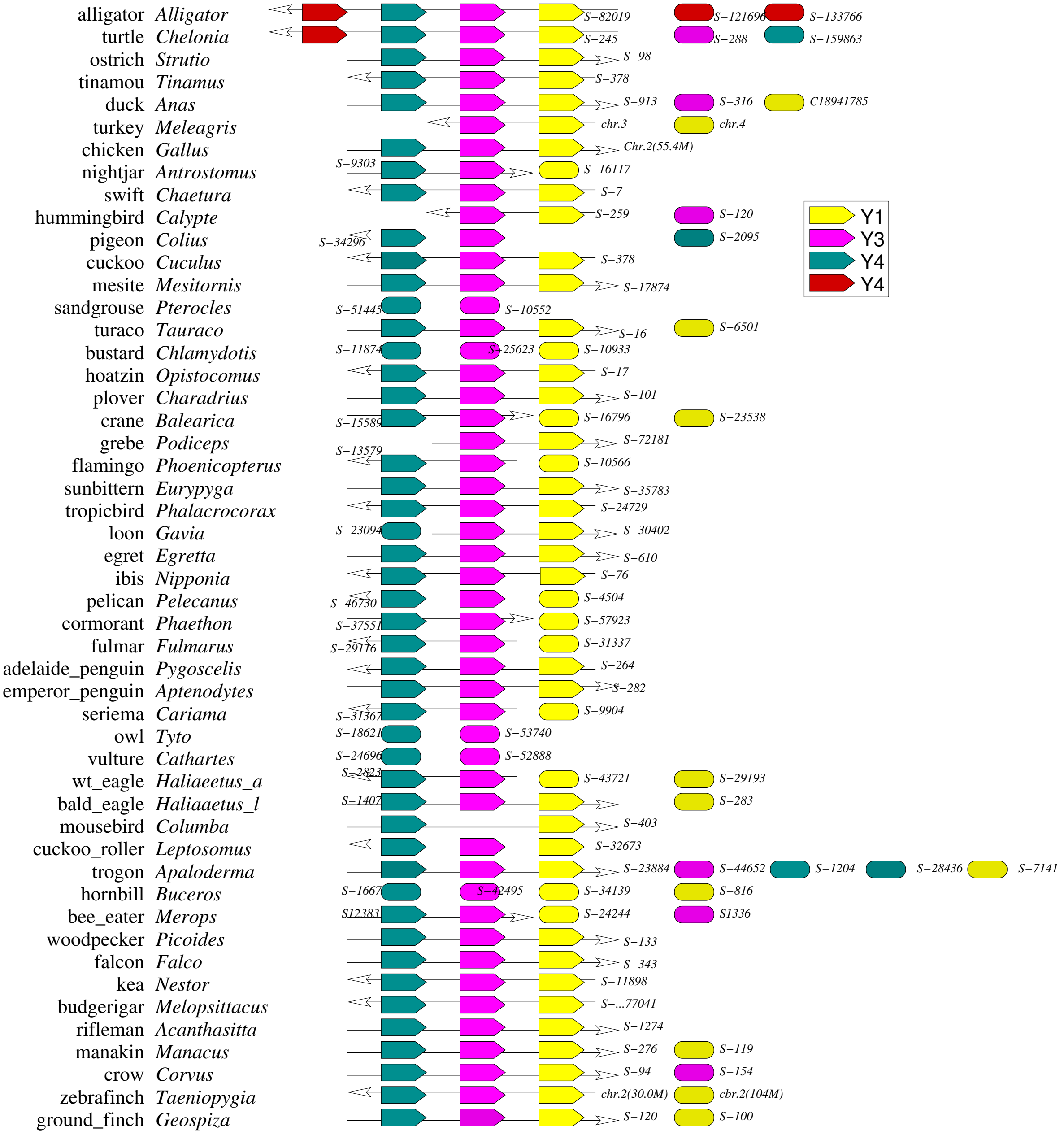

Figure E: Syntenic conservation of the Y RNA cluster in avian and reptile genomes.

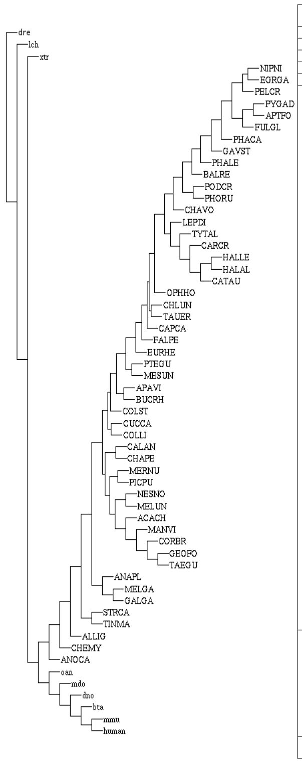

species	I-1 cluster						I-X cluster						II			
	mir-17	mir-18a	mir-19a	mir-20a	mir-19b-1	mir-92-1	mir-106a	mir-18b	mir-20b	mir-19b-2	mir-92-2	mir-363	mir-106b	mir-93	19-b-3	mir-25
dre	1	1	1	1	1	1	1	1	1	1	1	1	0	1	1	1
lch	1	1	1	1	1	1	1	1	1	1	1	1	0	1	0	1
xtr	1	1	1	1	1	1	1	1	1	1	1	1	0	1	0	1
NIPNI	1	1	1	1	1	1	1	1	1	1	1	1	0	0	0	0
EGRGA	1	1	1	1	1	1	1	1	1	1	1	1	0	0	0	0
PELCR	1	1	1	1	1	1	1	1	1	1	1	1	0	0	0	0
PYGAD	1	1	1	1	1	1	1	1	1	1	1	1	0	0	0	0
APTFO	1	1	1	1	1	1	1	1	1	1	1	1	0	0	0	0
FULGL	1	1	1	1	1	1	1	1	1	1	1	1	0	0	0	0
PHACA	1	1	1	1	1	1	1	1	1	1	1	1	0	0	0	0
GAVST	1	1	1	1	1	1	1	1	1	1	1	1	0	0	0	0
PHALE	1	1	1	1	1	1	1	1	1	1	1	1	0	0	0	0
BALRE	1	1	1	1	1	1	1	1	1	1	1	1	0	0	0	0
PODCR	1	1	1	1	1	1	1	1	1	1	1	1	0	0	0	0
PHORU	1	1	1	1	1	1	1	1	1	1	1	1	0	1	0	0
CHAVO	1	1	1	1	1	1	1	1	1	1	1	1	0	0	0	0
LEPDI	1	1	1	1	1	1	1	1	1	1	1	1	0	0	0	0
TYTAL	1	1	1	1	1	1	1	1	1	1	1	1	0	0	0	0
CARCR	1	1	1	1	1	1	1	1	1	1	1	1	0	0	0	0
HALLE	1	1	1	1	1	1	1	1	1	1	1	1	0	1	0	0
HALAL	1	1	1	1	1	1	1	1	1	1	1	1	0	0	0	0
CATAU	1	1	1	1	1	1	1	1	1	1	1	1	0	0	0	0
OPIHO	1	1	1	1	1	1	1	1	1	1	1	1	0	0	0	0
CHLUN	1	1	1	1	1	1	1	1	1	1	1	1	0	0	0	0
TAUER	1	1	1	1	1	1	1	1	1	1	1	1	0	0	0	0
ANTCA	1	1	1	1	1	1	1	1	1	1	1	1	0	0	0	0
FALPE	1	1	1	1	1	1	1	1	1	1	1	1	0	0	0	0
EURHE	1	1	1	1	1	1	1	1	1	1	1	1	0	0	0	0
PTEGU	1	1	1	1	1	1	1	1	1	1	1	1	0	0	0	0
MESUN	1	1	1	1	1	1	1	1	1	1	1	1	0	0	0	0
APAVI	1	1	1	1	1	1	1	1	1	1	1	1	0	0	0	0
BUCRH	1	1	1	1	1	1	1	1	1	1	1	1	0	0	0	0
COLST	1	1	1	1	1	1	1	1	1	1	1	1	0	0	0	0
CUCCA	1	1	1	1	1	1	1	1	1	1	1	1	0	0	0	0
COLLI	1	1	1	1	1	1	1	1	1	1	1	1	0	0	0	0
CALAN	1	1	1	1	1	1	1	1	1	1	1	1	0	0	0	0
CHAPE	1	1	1	1	1	1	1	1	1	1	1	1	0	0	0	0
MERNU	1	1	1	1	1	1	1	1	1	1	1	1	0	0	0	0
PICPU	1	1	1	1	1	1	1	1	1	1	1	1	0	0	0	0
NESNO	1	1	1	1	1	1	1	1	1	1	1	1	0	0	0	0
MELUN	1	1	1	1	1	1	1	1	1	1	1	1	0	0	0	0
ACACH	1	1	1	1	1	1	1	1	1	1	1	1	0	0	0	0
MANVI	1	1	1	1	1	1	1	1	1	1	1	1	0	0	0	0
CORBR	1	1	1	1	1	1	1	1	1	1	1	1	0	0	0	0
GEOFO	1	1	1	1	1	1	1	1	1	1	1	1	0	0	0	0
TAEGU	1	1	1	1	1	1	1	1	1	1	1	1	0	0	0	0
ANAPL	1	1	1	1	1	1	1	1	1	1	1	1	0	0	0	0
MELGA	1	1	1	1	1	1	1	1	1	1	1	1	0	0	0	0
GALGA	1	1	1	1	1	1	1	1	1	1	1	1	0	0	0	0
STRCA	1	1	1	1	1	1	1	1	1	1	1	1	0	0	0	0
TINMA	1	1	1	1	1	1	1	1	1	1	1	1	0	0	0	0
ALLIG	1	1	1	1	1	1	1	1	1	1	1	2	1	1	0	0
CHEMY	1	1	1	1	1	1	1	1	1	1	1	1	0	0	0	0
ANOCA	1	1	1	1	1	1	1	1	1	1	1	1	0	0	0	0
oan	2	1	1	1	1	1	1	1	1	1	1	1	0	0	0	0
mdo	1	1	1	1	1	1	1	1	1	1	1	1	0	1	0	1
dno	1	1	1	1	1	1	1	0	0	0	0	0	1	1	0	1
bta	1	1	1	1	1	1	1	1	1	1	1	1	1	1	0	1
mmu	1	1	1	1	1	1	1	1	1	1	1	1	1	1	0	1
HUMAN	1	1	1	1	1	1	1	1	1	1	1	1	1	1	0	1
species	mir-17	mir-18a	mir-19a	mir-20a	mir-19b-1	mr-92-1	mir-106a	mir-18b	mir-20b	mir-19b-2	mir-92-2		mir-106b	mir-93	19-b-3	mir-25
	I-1 cluster						I-X cluster						II			

Figure F: This figure illustrates the distribution of all members of the mir-17 clusters across birds, compared to fish and mammals. Columns correspond to single miRNA sequences, grouped by clusters and ordered by their position within the corresponding cluster. Rows correspond to the species and the cells contain the number of copies of the miRNA in the respective species. Colors correspond to orthologous miRNA families: mir-17 (orange), mir-19 (blue) and mir-92/25 (green).There are 3 clusters, I-1 and I-X, and cluster II. While the two copies of cluster I are completely conserved, cluster II has been lost as a whole in birds.

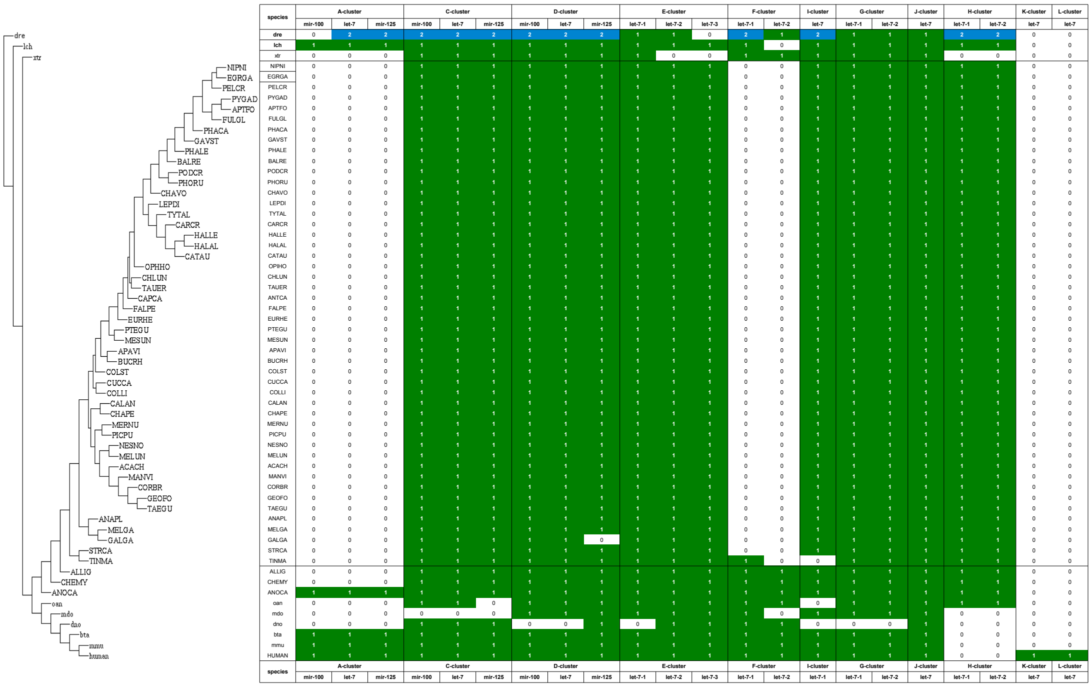

Figure G: This figure illustrates the distribution of the let-7 paralogs and clustered miRNAs of the families mir-100 and mir-125. Columns correspond to single miRNA sequences, grouped by clusters and ordered by their position within the corresponding cluster. Rows correspond to the species and the cells contain the number of copies of the miRNA in the respective species. Cluster A, which is strongly conserved in vertebrates has been completely lost in the avian lineage. Another obvious loss in birds is cluster F. However, in tinamou we detected an unclustered miRNA that best fits to F-let-7-1 in sequence and structure, while we could not find an I-let-7 miRNA sequence in this species.

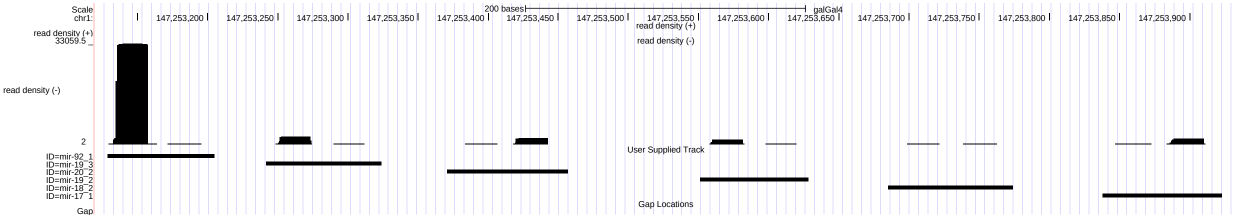

Figure H: microRNA 17 I-1 cluster: mir-92_1, mir-19_3, mir-20_2, mir-19_2, mir-18_2 & mir-17_1. The figure indicates the genomic location and the RNA-seq read-depths for these 6 microRNAs.

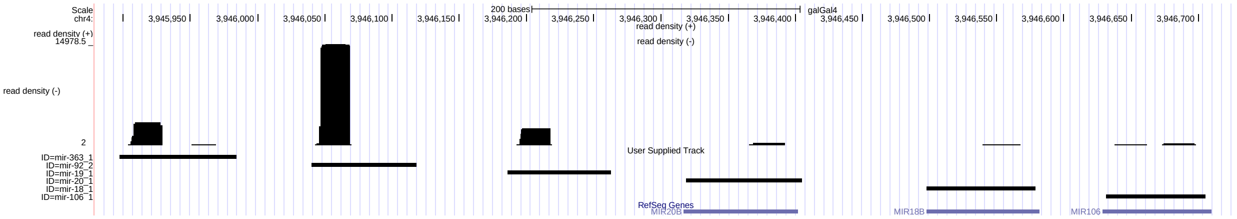

Figure I: microRNA 17 I-X cluster: mir-363_1, mir-92_2, mir-19_1, mir-20_1, mir-18_1, mir-106_1. The figure indicates the genomic location and the RNA-seq read-depths for these 6 microRNAs.

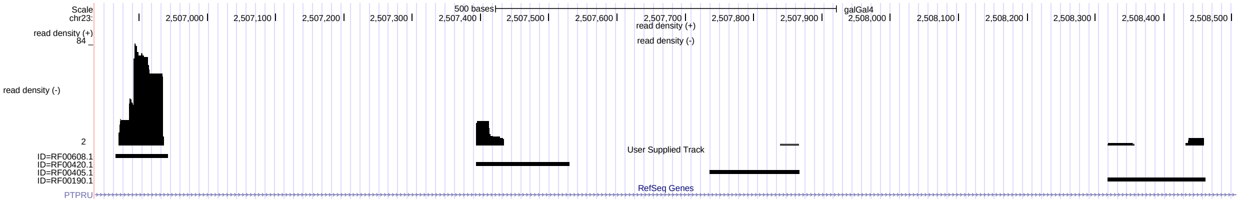

Figure J: H/ACA box snoRNA cluster: SNORA61, SNORA44, SNORA16. The figure indicates the genomic location and the RNA-seq read-depths for these 3 snoRNAs.

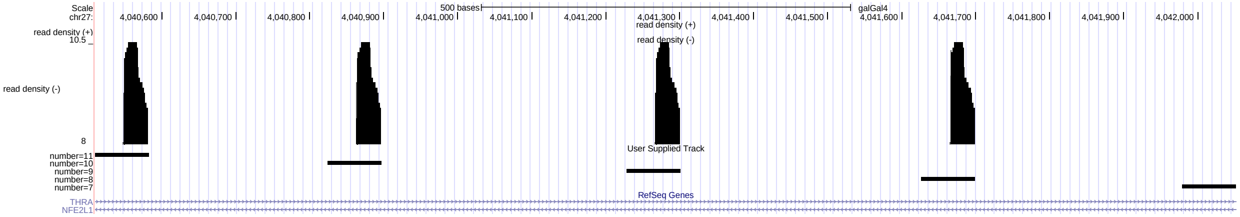

Figure K: Cysteine tRNA cluster. The figure indicates the genomic location and the RNA-seq read-depths for these 5 transfer RNAs.

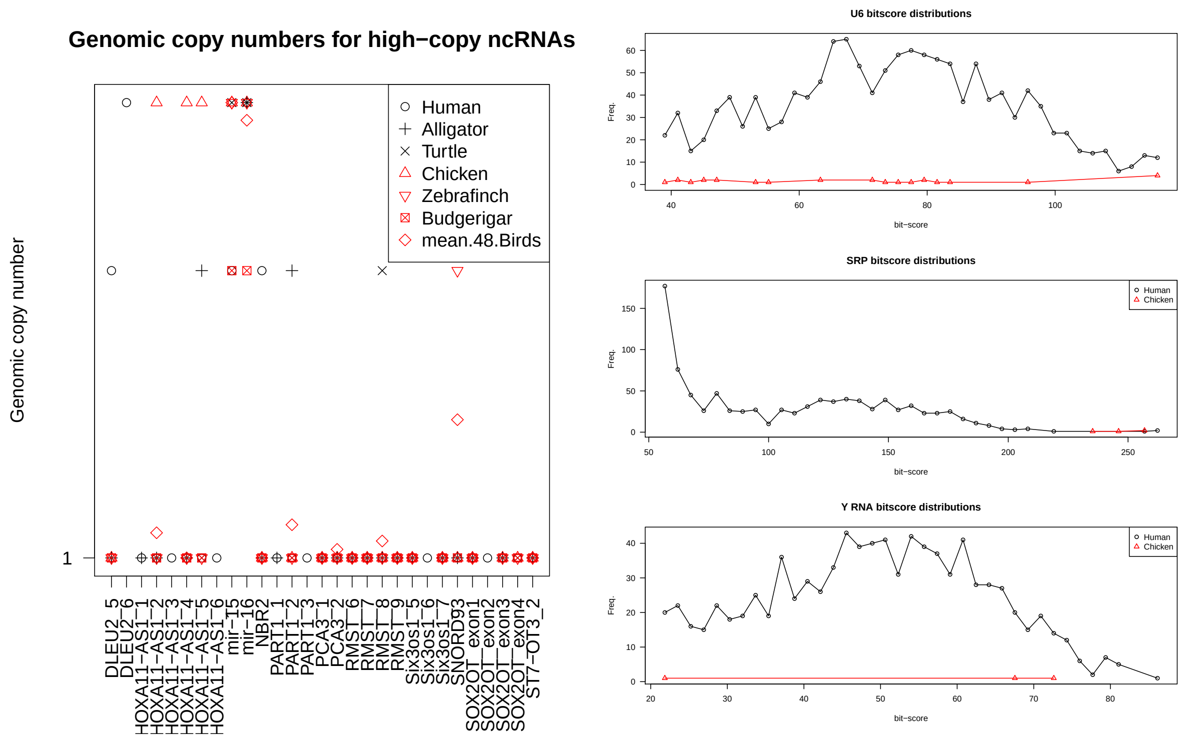

Figure L: **Left:** A comparison of the genomic copy numbers for 26 of the most abundant RNAs found in human, reptile and the bird genomes. **Right:** Bit-score distributions for the Rfam U6, Metazoan SRP and the Y RNA covariance models for human and chicken paralogues/pseudogenes. Loci with high bit-scores are more likely to be functional than those with low bit-scores (due to excess variation that conflicts with the canonical RNA models).

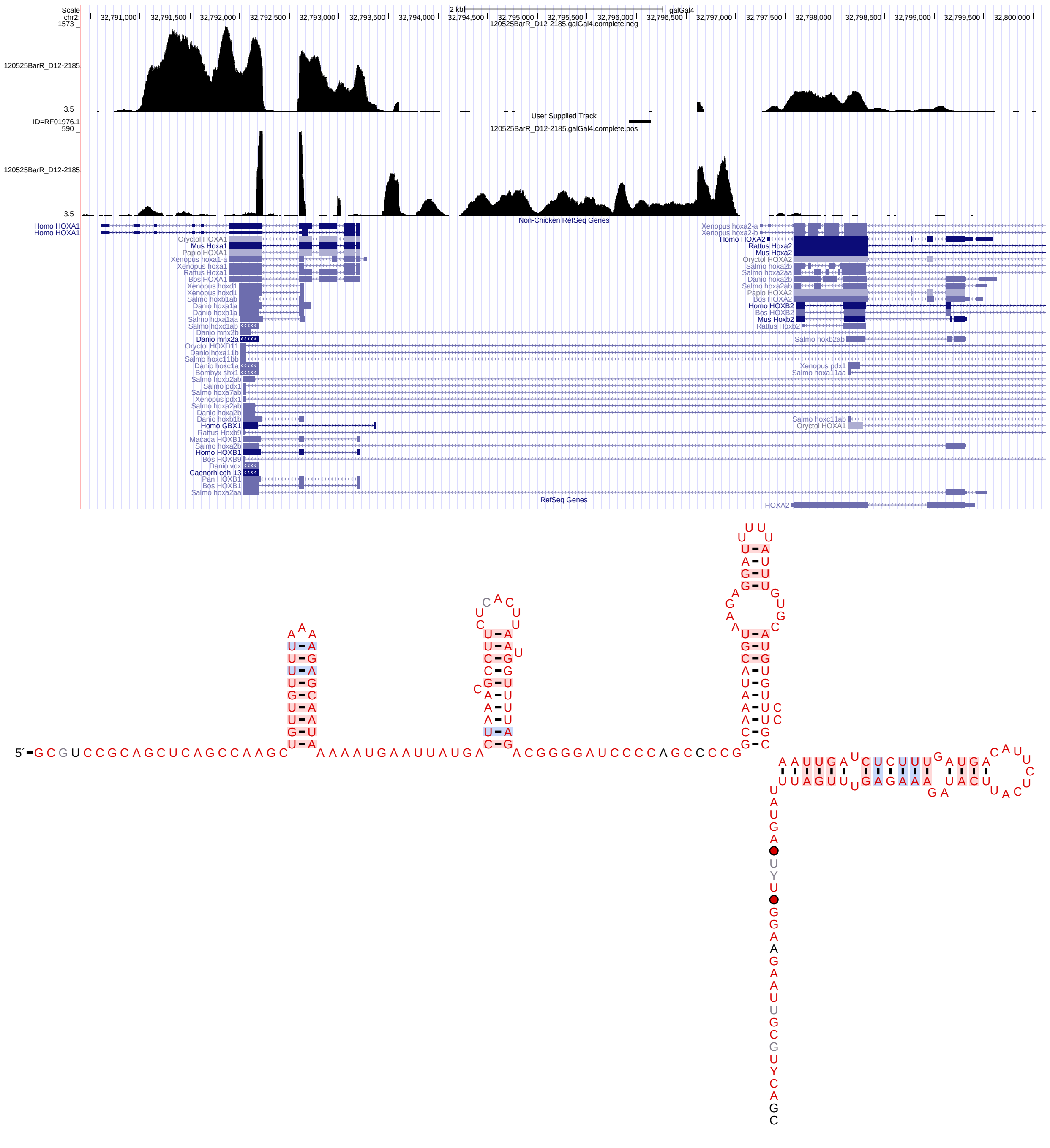

Figure M: Above: Expression of the chicken HOTAIRM1 (RF01976) locus and the surrounding genes: HOXA1, HOXA2, and HOXA3. Below: The predicted secondary structure of the Avian HOTAIRM1 domain.

RNA family (Rfam ID)	Chromosome	Coordinates	Strand
**Macro-chromosomes:**			
RNase MRP (RF00030)	chr4	39276202-39276488	-
**Micro-chromosomes:**			
U4atac snRNA (RF00618)	chr7	25702714-25702831	+
RNase P RNA (RF00009)	chr8	6609347-6609668	-
Telomerase RNA (RF00024)	chr9	19429028-19429084	+
Vault RNA (RF00006)	chr13	380747-380840	+
U11 snRNA (RF00548)	chr23	2216932-2217058	-

Table A: The location of "missing" RNA families in the chicken genome (galGal4).

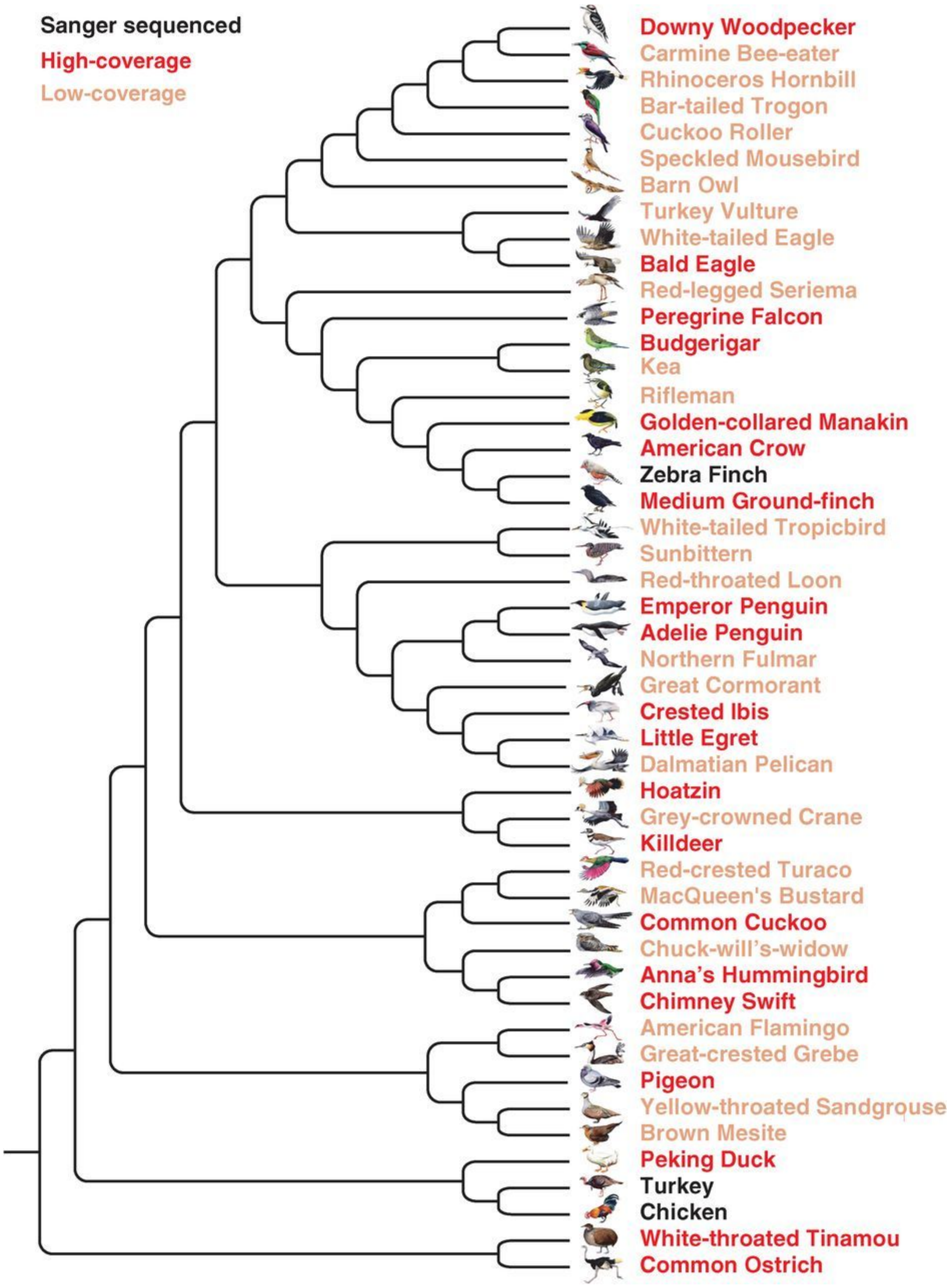

Figure N: The phylogenetic relationships between the Avian species used in this study. This figure has been reproduced, with permission from [15].

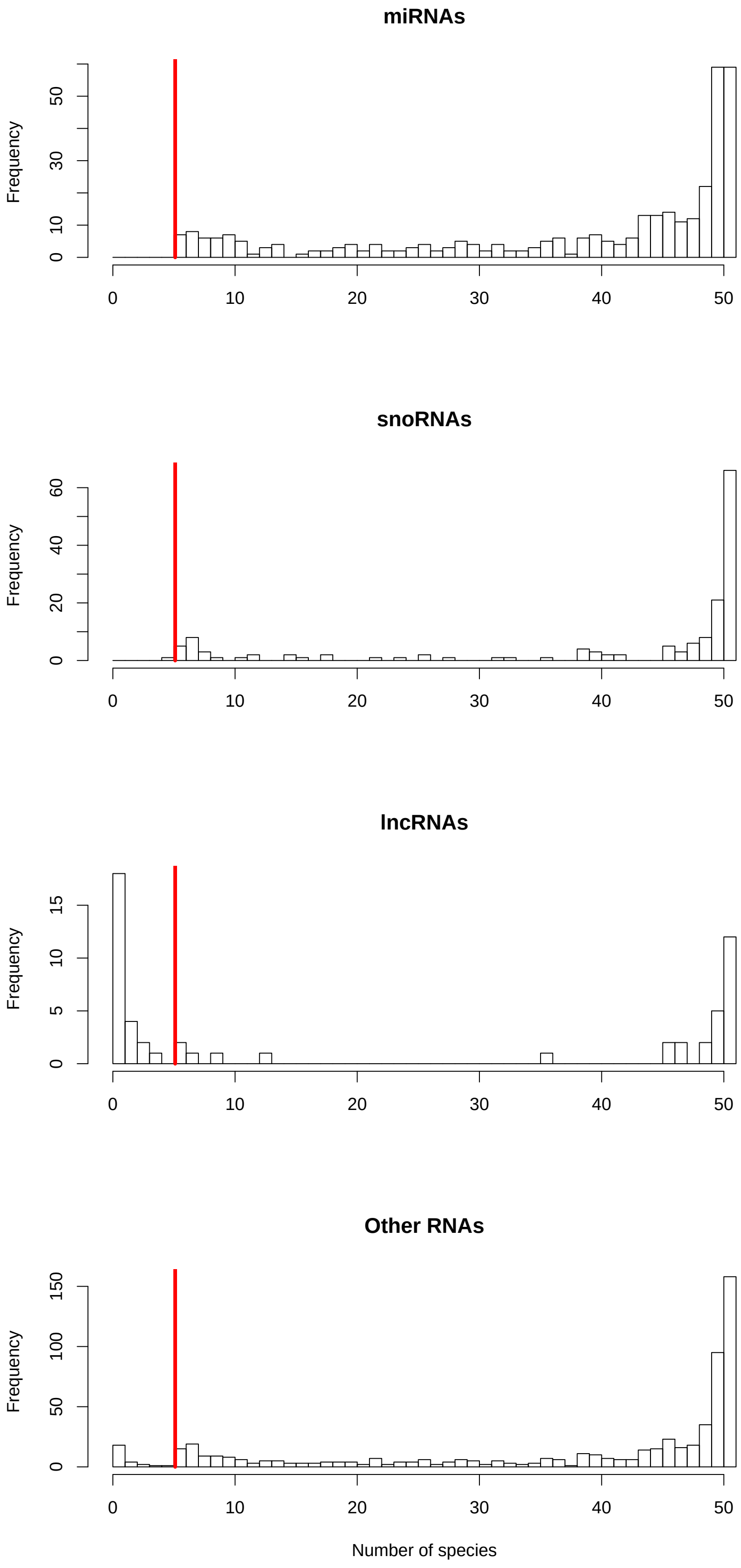

Figure O: The distribution of species counts for RNA families.

Database accessions

The NCBI BioProject, SRA and Study IDs for the genomes used in this study are are listed below.

Name	Species	BioProject ID	SRA ID	Study ID
Chimney Swift	*Chaetura pelagica*	PRJNA210808	SRA092327	SRP026688
Hummingbird	*Calypte anna*	PRJNA212866	SRA096094	SRP028275
Plover	*Charadrius vociferus*	PRJNA212867	SRA096158	SRP028286
Crow	*Corvus brachyrhynchos*	PRJNA212869	SRA096200	SRP028317
Cuckoo	*Cuculus canorus*	PRJNA212870	SRA096365	SRP028349
Manakin	*Manacus vitellinus*	PRJNA212872	SRA096507	SRP028393
Hoatzin	*Ophisthocomus hoazin*	PRJNA212873	SRA096539	SRP028409
Woodpecker	*Picoides pubescens*	PRJNA212874	SRA097131	SRP028625
Ostrich	*Struthio camelus*	PRJNA212875	SRA097407	SRP028745
Tinamou	*Tinamus guttatus*	PRJNA212876	SRA097796	SRP028753
Rifleman	*Acanthisitta chloris*	PRJNA212877	SRA097960	SRP028832
Trogon	*Apaloderma vittatum*	PRJNA212878	SRA097967	SRP028834
Crane	*Balearica regulorum*	PRJNA212879	SRA097970	SRP028839
Rhinoceros Hornbill	*Buceros rhinoceros*	PRJNA212887	SRA097991	SRP028845
Nightjar	*Antrostomus carolinensis*	PRJNA212888	SRA098079	SRP028883
Seriema	*Cariama cristata*	PRJNA212889	SRA098089	SRP028884
Turkey Vulture	*Cathartes aura*	PRJNA212890	SRA098145	SRP028913
Bustard	*Chlamydotis macqueenii*	PRJNA212891	SRA098203	SRP028950
Mousebird	*Colius striatus*	PRJNA212892	SRA098342	SRP028965
Sunbittern	*Eurypyga helias*	PRJNA212893	SRA098749	SRP029147
Northern Fulmar	*Fulmarus glacialis*	PRJNA212894	SRA098806	SRP029180
Red-throated Loon	*Gavia stellata*	PRJNA212895	SRA098829	SRP029187
White-tailed Eagle	*Haliaeetus albicilla*	PRJNA212896	SRA098868	SRP029203
Bald Eagle	*Haliaeetus leucocephalus*	PRJNA237821<br>SRX475900<br>SRX475901<br>SRX475902	SRX475899	SRP038924
Cuckoo Roller	*Leptosomus discolor*	PRJNA212897	SRA098894	SRP029206
Bee-eater	*Merops nubicus*	PRJNA212898	SRA099305	SRP029278
Brown Mesite	*Mesitornis unicolor*	PRJNA212899	SRA099409	SRP029309
Kea	*Nestor notabilis*	PRJNA212900	SRA099410	SRP029311
Pelican	*Pelecanus crispus*	PRJNA212901	SRA099411	SRP029331
Tropicbird	*Phaethon lepturus*	PRJNA212902	SRA099412	SRP029342
Cormorant	*Phalacrocorax carbo*	PRJNA212903	SRA099413	SRP029344
Flamingo	*Phoenicopterus ruber*	PRJNA212904	SRA099414	SRP029345
Grebe	*Podiceps cristatus*	PRJNA212905	SRA099415	SRP029346
Sandgrouse	*Pterocles gutturalis*	PRJNA212906	SRA099416	SRP029347
Turaco	*Tauraco erythrolophus*	PRJNA212908	SRA099418	SRP029348
Barn Owl	*Tyto alba*	PRJNA212909	SRA099419	SRP029349
Crested Ibis	*Nipponia nippon*	PRJNA232572	SRA122361	SRP035852
Little Egret	*Egretta garzetta*	PRJNA232959	SRA123137	SRP035853

Table B: The NCBI BioProject/SRA and Study IDs used in this study.

Common name	Species	BioProject ID	SRA ID	Study ID
Emperor Penguin	*Aptenodytes forsteri*	PRJNA235982	SRA129317	SRP035855
Adelie Penguin	*Pygoscelis adeliae*	PRJNA235983	SRA129318	SRP035856
Chicken	*Gallus gallus*	PRJNA13342	SRA030184	SRP005856 (galGal4)
Zebra Finch	*Taeniopygia guttata*	PRJNA17289	SRA010067	SRP001389
Turkey	*Meleagris gallopavo*	PRJNA42129	Unknown	Unknown
Budgerigar	*Melopsittacus undulatus*	PRJEB1588	ERA200248	ERP002324
Mallard	*Anas platyrhynchos*	PRJNA46621	SRA010308	SRP001571
Rock Pigeon	*Columba livia*	PRJNA167554	SRA054954	SRP013894
Peregrine Falcon	*Falco peregrinus*	PRJNA159791	SRA055082	SRP013939
Medium Ground-finch	*Geospiza fortis*	PRJNA156703	SRA051234	SRP011940
Outgroups				
Human	*Homo sapiens*			HG19/GRCh37
Alligator	*Alligator mississippiensis*			allMis1
Green Turtle	*Chelonia mydas*	PRJNA104937		
RNA-seq data				
Chicken	*Gallus gallus*	PRJNA204941	NA	NA
Chicken	*Gallus gallus*	NA	NA	SRP041863

Table C: The NCBI BioProject/SRA and Study IDs for the previously published genomes used in this study.

References

1. Xie M, Mosig A, Qi X, Li Y, Stadler PF, Chen JJL: **Size Variation and Structural Conservation of Vertebrate Telomerase RNA**. *J. Biol. Chem.* 2008, **283**:2049–2059.
2. Stadler PF, Chen JJL, Hackermüller J, Hoffmann S, Horn F, Khaitovich P, Kretzschmar AK, Mosig A, Prohaska SJ, Qi X, Schutt K, Ullmann K: **Evolution of Vault RNAs**. *Mol. Biol. Evol.* 2009, **26**:1975–1991.
3. Kolbe DL, Eddy SR: **Local RNA structure alignment with incomplete sequence.** *Bioinformatics* 2009, **25**(10):1236–43.
4. Bejerano G, Pheasant M, Makunin I, Stephen S, Kent WJ, Mattick JS, Haussler D: **Ultraconserved elements in the human genome.** *Science* 2004, **304**(5675):1321–5.
5. Bejerano G, Lowe CB, Ahituv N, King B, Siepel A, Salama SR, Rubin EM, Kent WJ, Haussler D: **A distal enhancer and an ultraconserved exon are derived from a novel retroposon.** *Nature* 2006, **441**(7089):87–90.
6. Braconi C, Valeri N, Kogure T, Gasparini P, Huang N, Nuovo GJ, Terracciano L, Croce CM, Patel T: **Expression and functional role of a transcribed noncoding RNA with an ultraconserved element in hepatocellular carcinoma.** *Proc Natl Acad Sci U S A* 2011, **108**(2):786–91.
7. Lerner MR, Boyle JA, Hardin JA, Steitz JA: **Two novel classes of small ribonucleoproteins detected by antibodies associated with lupus erythematosus.** *Science* 1981, **211**(4480):400–2.
8. Christov CP, Gardiner TJ, Szüts D, Krude T: **Functional requirement of noncoding Y RNAs for human chromosomal DNA replication.** *Mol Cell Biol* 2006, **26**(18):6993–7004.
9. Mosig A, Guofeng M, Stadler BM, Stadler PF: **Evolution of the vertebrate Y RNA cluster.** *Theory Biosci* 2007, **126**:9–14.
10. Kong LB, Siva AC, Rome LH, Stewart PL: **Structure of the vault, a ubiquitous celular component.** *Structure* 1999, **7**(4):371–9.
11. Stadler PF, Chen JJ, Hackermüller J, Hoffmann S, Horn F, Khaitovich P, Kretzschmar AK, Mosig A, Prohaska SJ, Qi X, Schutt K, Ullmann K: **Evolution of vault RNAs.** *Mol Biol Evol* 2009, **26**(9):1975–91.
12. Cabili MN, Trapnell C, Goff L, Koziol M, Tazon-Vega B, Regev A, Rinn JL: **Integrative annotation of human large intergenic noncoding RNAs reveals global properties and specific subclasses.** *Genes Dev* 2011, **25**(18):1915–27.

13. Kutter C, Watt S, Stefflova K, Wilson MD, Goncalves A, Ponting CP, Odom DT, Marques AC: **Rapid turnover of long noncoding RNAs and the evolution of gene expression.** *PLoS Genet* 2012, **8**(7):e1002841.

14. Hoeppner MP, Gardner PP, Poole AM: **Comparative analysis of RNA families reveals distinct repertoires for each domain of life.** *PLoS Comput Biol* 2012, **8**(11):e1002752.

15. Jarvis ED, Mirarab S, Aberer AJ, Li B, Houde P, Li C, Ho SYW, Faircloth BC, Nabholz B, Howard JT, Suh A, Weber CC, da Fonseca RR, Li J, Zhang F, Li H, Zhou L, Narula N, Liu L, Ganapathy G, Boussau B, Bayzid MS, Zavidovych V, Subramanian S, Gabaldón T, Capella-Gutiérrez S, Huerta-Cepas J, Rekepalli B, Munch K, Schierup M, Lindow B, Warren WC, Ray D, Green RE, Bruford MW, Zhan X, Dixon A, Li S, Li N, Huang Y, Derryberry EP, Bertelsen MF, Sheldon FH, Brumfield RT, Mello CV, Lovell PV, Wirthlin M, Schneider MPC, Prosdocimi F, Samaniego JA, Velazquez AMV, Alfaro-Núñez A, Campos PF, Petersen B, Sicheritz-Ponten T, Pas A, Bailey T, Scofield P, Bunce M, Lambert DM, Zhou Q, Perelman P, Driskell AC, Shapiro B, Xiong Z, Zeng Y, Liu S, Li Z, Liu B, Wu K, Xiao J, Yinqi X, Zheng Q, Zhang Y, Yang H, Wang J, Smeds L, Rheindt FE, Braun M, Fjeldsa J, Orlando L, Barker FK, Jønsson KA, Johnson W, Koepfli KP, O'Brien S, Haussler D, Ryder OA, Rahbek C, Willerslev E, Graves GR, Glenn TC, McCormack J, Burt D, Ellegren H, Alström P, Edwards SV, Stamatakis A, Mindell DP, Cracraft J, Braun EL, Warnow T, Jun W, Gilbert MTP, Zhang G: **Whole-genome analyses resolve early branches in the tree of life of modern birds**. *Science* 2014, **346**(6215):1320–1331.
